# CTLA-4 gene polymorphisms and risk of idiopathic recurrent pregnancy loss in a Tunisian population

**DOI:** 10.1186/1471-2164-15-S2-P11

**Published:** 2014-04-02

**Authors:** Safia Messaoudi, Ilham Houas, Khadijah Yaseen, Mariem Dandana, Touhami Mahjoub

**Affiliations:** 1 Laboratory of Human Genome and Multifactorial Diseases, Faculty of Pharmacy of Monastir, University of Monastir, Tunisia

## Background

An aberrant regulation of immunological, metabolic, vascular and endocrine processes leads to obstetric complications, including recurrent pregnancy loss (RPL) [1]. Cytotoxic T lymphocyte-associated antigen 4 (CTLA-4) is a negative regulator of T cell activation [2] expressed in placental fibroblasts and may modulate peripheral self-tolerance of the allogenic fetus [3,4]. It is a stimulatory molecule involved in T-cell activation at the maternal–fetal interface in women with unexplained pregnancy loss [5,6]. In this study, we investigate the possible associations of Cytotoxic T lymphocyte-associated antigen 4 (CTLA-4) gene polymorphisms with idiopathic recurrent pregnancy loss (RPL).

## Materials and methods

We investigated the association of the CTLA-4 gene single nucleotide polymorphisms (SNPs) -318 C/T (rs5742909), +49A/G (rs231775), and CT60 A/G (rs3087243), by TaqMan assays in analysis in 470 Tunisian women comprising 235 RPL cases and 235 multi-parous controls. The association of CTLA-4 alleles, genotypes, and haplotypes with RPL was evaluated by Fisher's exact test and regression analysis.

## Results

The CTLA-4 variants rs5742909, rs231775, and rs3087243, were in Hardy Weinberg equilibrium, and low linkage disequilibrium was noted between the three studied SNPs. The frequency of rs231775 G allele (P=0.04), but not rs3087243 G allele (P=0.61) or rs5742909 T allele (P=1), was higher in RPL cases than in control women. Significant differences in the distribution of rs231775 (P<0.02), but not rs5742909 (P=0.21) or rs3087243 (P=0.49) genotypes were seen between cases and controls, and only rs231775 showed a significant association with RPL, with increments of 1.74 in disease risk seen for heterozygous carriers. Only rs231775 (+49/A/G), previously shown associated with the immuno-pathogenesis of RPL, and it confers susceptibility to RPL in Chinese population [5] and North Indian Women [7], was significantly associated with RPL. Among the six three-locus CTLA-4 haplotypes constructed (rs5742909/rs231775/rs3087243), increased frequency of CGA (P=0.0046), and CAG (P=0.036) haplotypes were seen in RPL cases, thus conferring disease susceptibility nature to these haplotypes.

## Conclusions

The rs231775 AG genotype and CGA and CAG haplotypes may contribute to RPL development in a Tunisian Population.
